# Supplementary material for: NMR characterization of RNA binding property of the DEAD-box RNA helicase DDX3X and its implications for helicase activity
Source: Nat Commun. 2024 Apr 25;15:3303. doi: 10.1038/s41467-024-47659-w (PMC11045745; doi:10.1038/s41467-024-47659-w)
Supplement: Supplementary file 1 — Supplementary Information [file 41467_2024_47659_MOESM1_ESM.pdf]

Supplementary Information

NMR characterization of RNA binding property of the DEAD-box RNA helicase DDX3X and its implications for helicase activity

Yuki Toyama[1,*] and Ichio Shimada[1,2,*]

1. RIKEN Center for Biosystems Dynamics Research (BDR), 1-7-22 Suehiro-cho, Tsurumi-ku, Yokohama, Kanagawa 230-0045, Japan

2. Graduate School of Integrated Sciences for Life, Hiroshima University, 1-4-4 Kagamiyama, Higashi-Hiroshima, Hiroshima 739-8528, Japan

*Address correspondence to: yuki.toyama@riken.jp, ichio.shimada@riken.jp

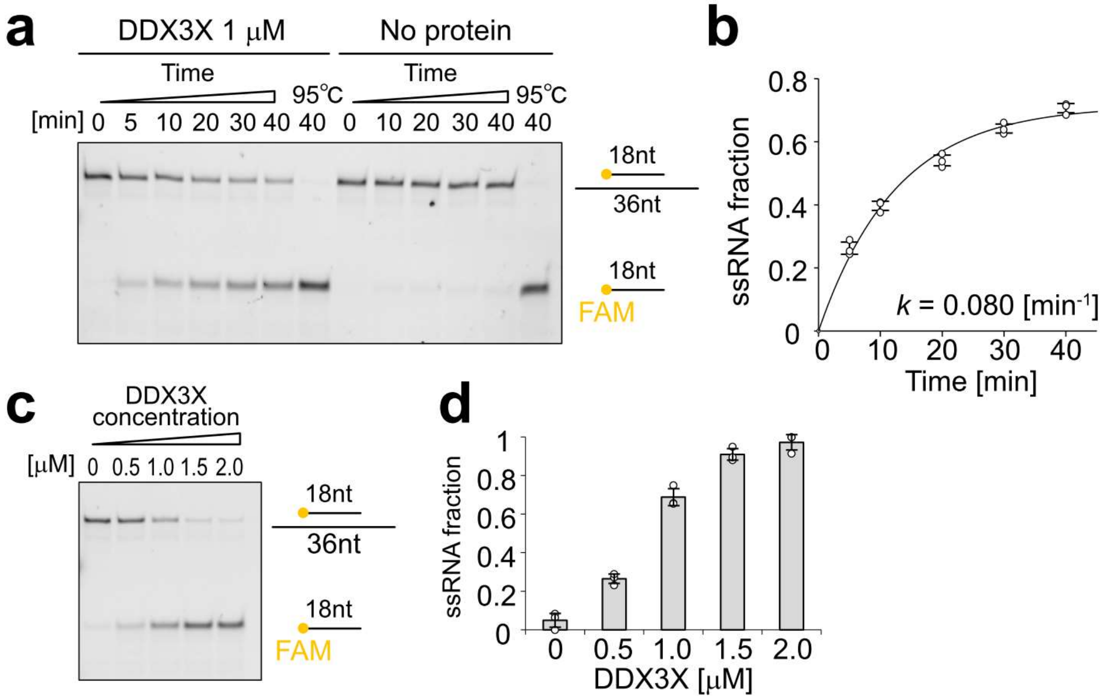

Supplementary Figure 1 Unwinding assays of DDX3X. (a) Unwinding reactions were monitored as a function of incubation time in the presence or absence of 1 μM DDX3X. Control samples were prepared by heating at 95 °C for 3 minutes to completely displace the 18mer/36mer dsRNA. (b) Plot of the ssRNA fraction as a function of incubation time. The apparent rate constant, k, for the unwinding reaction was obtained by fitting the data to a function, $y = a \times \exp(-kt) + b$, where a and b are the constants. Error bars represent the standard deviation of the three independent measurements (n=3). The center of the error bar represents the average value. (c) Unwinding reaction mixtures with varying concentrations of DDX3X after 30 mins of incubation were analyzed. (d) Plots of the ssRNA fraction as a function of DDX3X concentration. Error bars represent the standard deviation of three independent measurements (n=3). The center of the error bar represents the average value. All of the unwinding assays were performed at 37 °C. Source data are provided as a Source Data file.

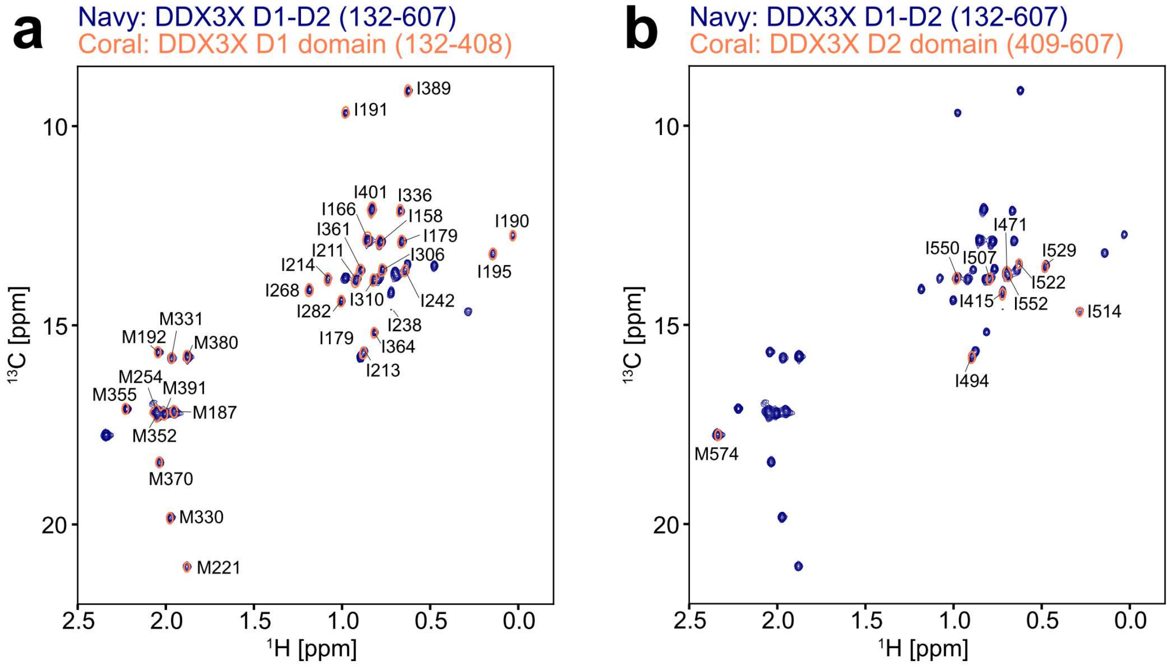

Supplementary Figure 2 NMR spectra and assignments of DDX3X and its individual domains. (a) Overlay of the ^{13}C-^{1}H HMQC spectra of [U-^{2}H; Ileδ1-^{13}C^{1}H$_3$; Metε-^{13}C^{1}H$_3$]-labeled DDX3X (navy, multiple contours) and [U-^{2}H; Ileδ1-^{13}C^{1}H$_3$; Metε-^{13}C^{1}H$_3$]-labeled D1 domain (coral, single contour). Assignments of the methyl groups from the D1 domain are shown. (b) Overlay of the ^{13}C-^{1}H HMQC spectra of [U-^{2}H; Ileδ1-^{13}C^{1}H$_3$; Metε-^{13}C^{1}H$_3$]-labeled DDX3X (navy, multiple contours) and [U-^{2}H; Ileδ1-^{13}C^{1}H$_3$; Metε-^{13}C^{1}H$_3$]-labeled D2 domain (coral, single contour). Assignments of the methyl groups from the D2 domain are shown. NMR measurements were performed at 35 °C and 900 MHz (for DDX3X) or 1 GHz (for D1 and D2 domains). The M379 signal was not observed in the spectrum.

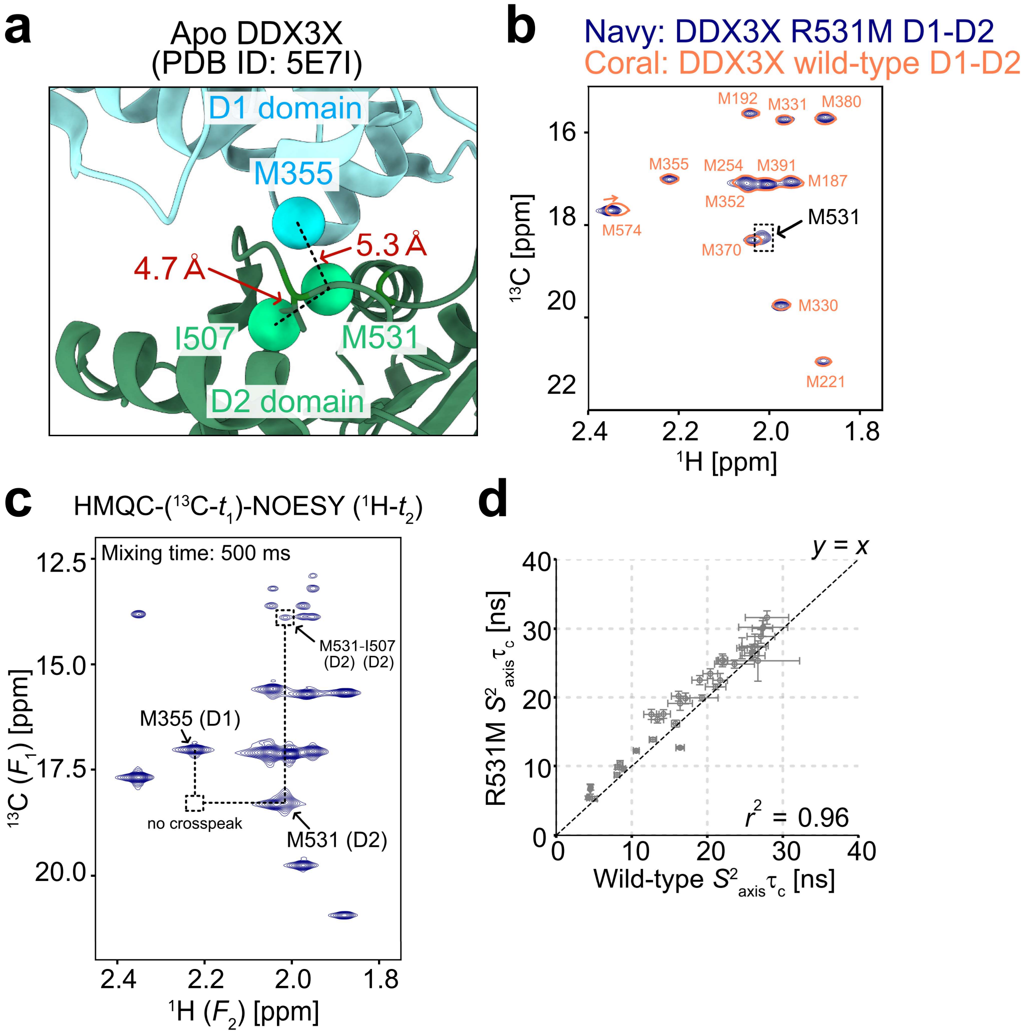

Supplementary Figure 3 Interdomain interaction of DDX3X. (a) Close-up view of the interface formed between the D1 and D2 domains of DDX3X in the apo state (PDB ID: 5E7I). R531 was replaced with Met by using the ChimeraX *swapaa* module. The Cε-Cε distance between M355 and M531 and the Cε-Cδ1 distance between M531 and I507 are displayed. (b) Overlay of the ^{13}C-^{1}H HMQC spectra of [u-^{2}H; Ileδ1-^{13}C^{1}H$_3$; Metε-^{13}C^{1}H$_3$]-labeled wild-type (coral, single contour) and [U-^{2}H; Ileδ1-^{13}C^{1}H$_3$; Met αβγ-^{2}H, ε-^{13}C^{1}H$_3$]-labeled R531M variant (navy, multiple contours) DDX3X. The spectrum of the wild type was shifted by 0.1 ppm in the ^{13}C dimension to correct for the isotope shift. (c) HMQC-(^{13}C-t_1)-NOESY (^{1}H-t_2) (500 ms mixing time) spectrum of the R531M variant DDX3X. (d) Correlation plot of the $S^2_{\mathrm{axis}}\tau_C$ values of wild-type and R531M variant DDX3X. The error bars represent the uncertainties of the fitted parameters as estimated from the covariance matrix. r^2 is Pearson's correlation coefficient squared. Source data are provided as a Source Data file.

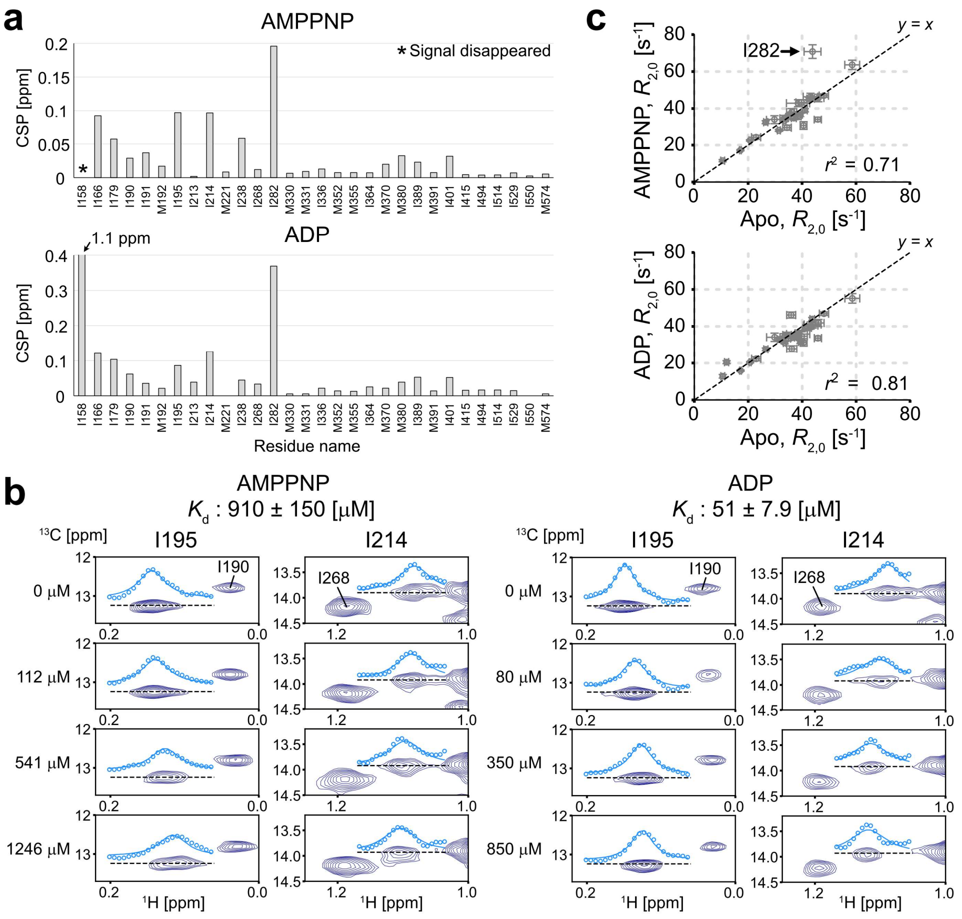

Supplementary Figure 4 Interactions of DDX3X with AMPPNP or ADP. (a) Plots of the normalized chemical shift difference upon the addition of 1.5 mM AMPPNP (top) or 1 mM ADP (bottom). The normalized chemical shift perturbation value (CSP) was calculated as $\Delta\delta = \sqrt{(\Delta\delta_H/\alpha)^2 + (\Delta\delta_C/\beta)^2}$, where $\Delta\delta_{\mathrm{H}}$ and $\Delta\delta_{\mathrm{C}}$ are shift differences in ^{1}H and ^{13}C dimensions, and α and β are standard deviations of ^{1}H and ^{13}C chemical shift distributions deposited in the Biological Magnetic Resonance Data Bank (α = 0.28, β = 1.634 for Ile, and α = 0.377, β = 1.701 for Met). (b) Two-dimensional lineshape analyses of I195 and I214 methyl signals to obtain the dissociation constants (K_{d}) for AMPPNP (left) and ADP (right). Experimental ^{1}H 1D slices (dots) and the fitted values (lines) are shown. All NMR datasets were recorded at 600 MHz and 35 °C. The error of the K_{d} value was estimated using the jackknife method. (c) Correlation plots of the apparent transverse relaxation rates of methyl ^{13}C ($R_{2,0}$) measured with and without AMPPNP (top) or ADP (bottom). The error bars represent the uncertainties of the fitted parameters as estimated from the covariance matrix. The outlier, I282, is located in the ATP-binding pocket (see Fig. 1d in the main text). r^2 is Pearson's correlation coefficient squared. Source data are provided as a Source Data file.

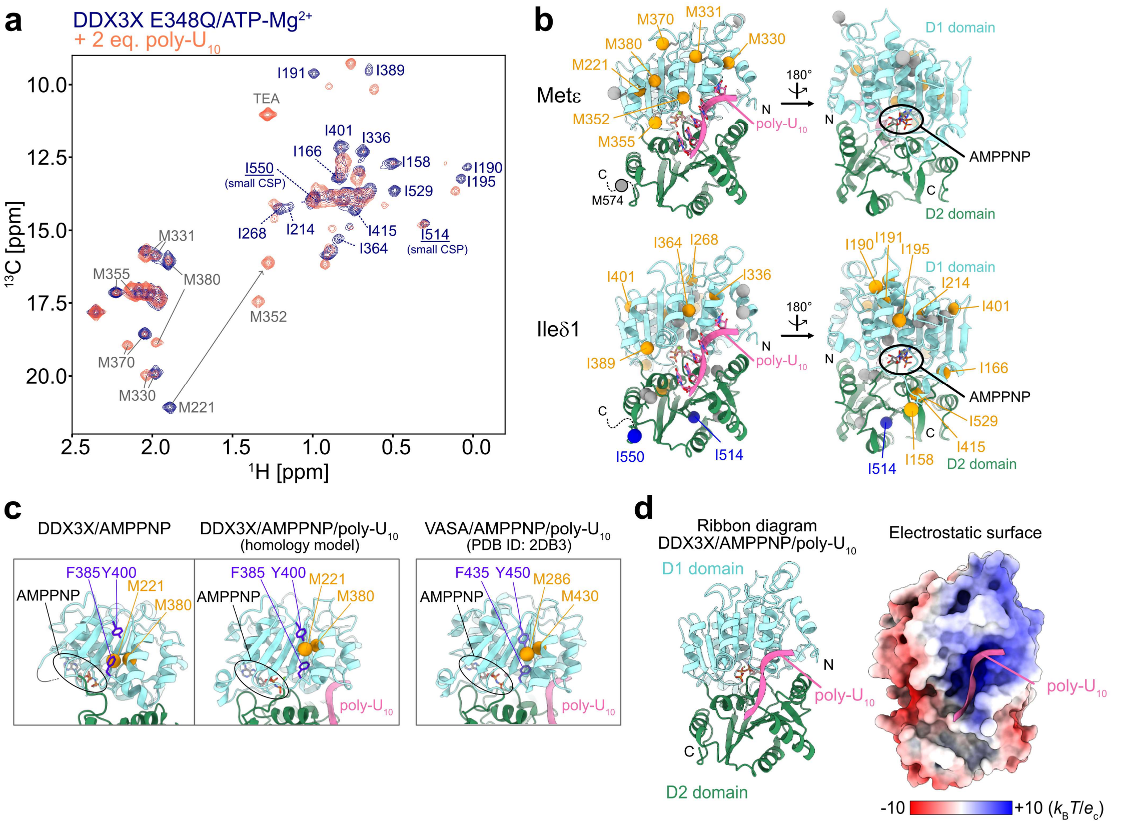

Supplementary Figure 5 Interaction of the E348Q variant DDX3X with poly-U$_{10}$. (a) Ile and Met methyl region of the ^{13}C-^{1}H HMQC spectra of the E348Q variant DDX3X with (orange-red) and without (navy) 2 equimolar poly-U$_{10}$. The signal at ^{1}H and ^{13}C chemical shift of 1.28 ppm and 11.0 ppm is from triethylamine (TEA), a counter-ion of the poly-U$_{10}$ RNA. The assignments of the Met methyl signal that showed a significant chemical shift difference are shown. NMR measurements were performed at 35 °C and 600 MHz in the presence of 5 mM ATP/$MgCl_2$. (b) Mapping of Met (top) and Ile (bottom) residues that showed significant chemical shift changes upon binding to poly-U$_{10}$ onto the modeled structure of DDX3X/AMPPNP/poly-U$_{10}$. Met Cε and Ile Cδ1 carbons are shown as spheres. The Met methyl probes that showed significant chemical shift perturbation (CSP) are colored orange, while those with small or undefined CSP are colored gray. M574 is located in the C-terminal tail region whose structure was not modeled from the VASA/AMPPNP/poly-U$_{10}$ crystal structure (PDB ID: 2DB3). Since the complete set of assignments for Ile methyl probes in the poly-U$_{10}$ bound state was not available due to severe signal overlaps and broadenings, we analyzed the chemical shift perturbation of Ile methyl probes by examining the disappearance of the free state signals. The Ile methyl probes with large CSP and/or marked intensity reduction are colored orange, while those with small CSP are colored blue.

The methyl probes with undefined CSP are colored gray. (c) Close-up view of the structure of the hydrophobic cluster located between the AMPPNP and poly-U_{10} binding sites. The side-chain methyl groups of M221 and M380, as well as the nearby aromatic residues, are highlighted. The structure of the DDX3X/AMPPNP complex (PDB ID: 5E7M) (left), the homology model of DDX3X/AMPPNP in complex with poly-U_{10} (center), and the structure of VASA/AMPPNP/poly-U_{10} complex (PDB ID: 2DB3) (right) are shown. The highlighted Met residues and aromatic residues are well conserved. (d) Ribbon diagram (left) and the electrostatic surface (right) of the homology model of DDX3X/AMPPNP in complex with poly-U_{10}.

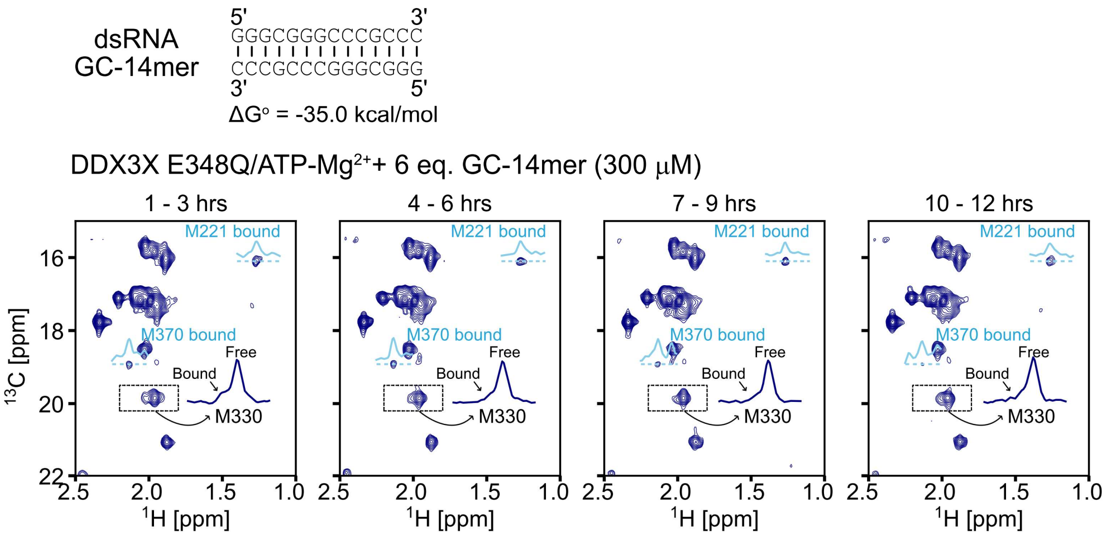

Supplementary Figure 6 Equilibrium condition of the DDX3X-GC-14mer dsRNA interaction. ^{13}C-^{1}H HMQC spectra of [Frac-^{2}H; Ileδ1-$^{13}C^{1}H_3$; Metε-$^{13}C^{1}H_3$]-labeled DDX3X E348Q (50 μM) in the presence of 6 eq. (300 μM) GC-14mer dsRNA were recorded every 3 hours. The 1D slices of the bound state signal of M221 and M370 are shown in light blue. The 1D projections of the dotted region containing the free and bound signals for M330 are shown in each spectrum. Duplex stability (ΔG°) at 35 °C was estimated by using nearest-neighbor parameters in 1 M NaCl. All NMR measurements were performed at 35 °C and 600 MHz.

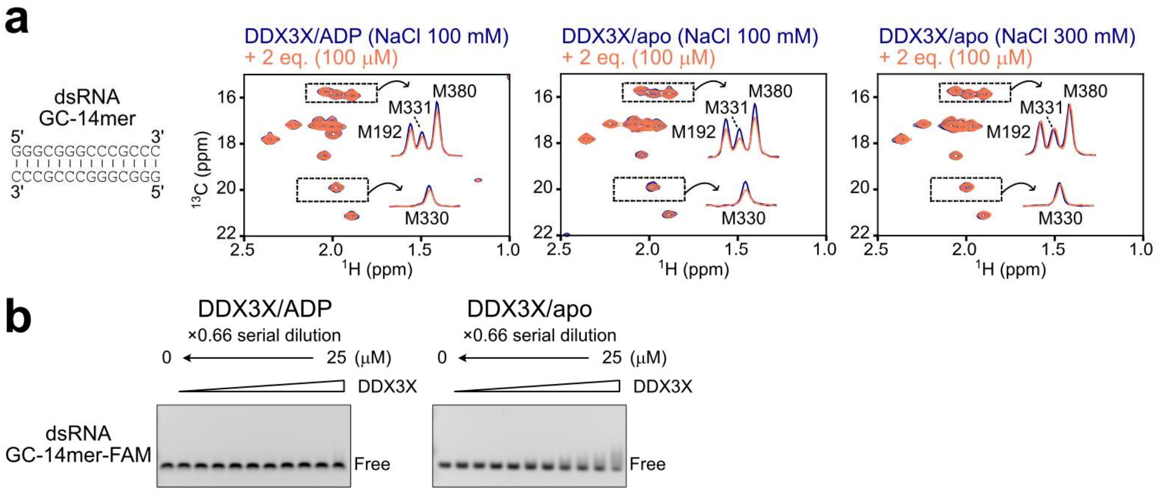

Supplementary Figure 7 Interaction of DDX3X with GC-14mer dsRNA. (a) Met methyl region of the ^{13}C-^{1}H HMQC spectra of the wild-type DDX3X in the ADP-bound state with 100 mM NaCl (left), the apo state with 100 mM NaCl (center), and the apo state with 300 mM NaCl (right). The spectra measured in the absence (navy) or presence (orange-red) of 2 equimolar (as a single strand) 14mer dsRNA are shown. The protein concentration was 20 μM (for apo DDX3X with 100 mM NaCl) or 50 μM (for the others). The signal intensities were normalized according to the protein concentration. The 1D projections of the dotted regions are shown in each spectrum. Slightly larger intensity reductions were observed in the apo-state with 100 mM NaCl (center), suggesting the presence of a weak interaction with dsRNA in the apo state. Since the interaction was efficiently suppressed by adding 300 mM NaCl (right), the interaction observed at the lower NaCl concentration is likely due to the weak electrostatic interaction between DDX3X and dsRNA which can be more significant in the absence of ATP/ADP nucleotides in the solution. NMR measurements were performed at 35 °C and 600 MHz. (b) EMSA binding experiments for ADP-bound (left) or apo (right) DDX3X using GC-14mer-FAM dsRNA. DDX3X protein was titrated from 0 to 25 μM. The binding experiments were repeated twice with similar results. Source data are provided as a Source Data file.

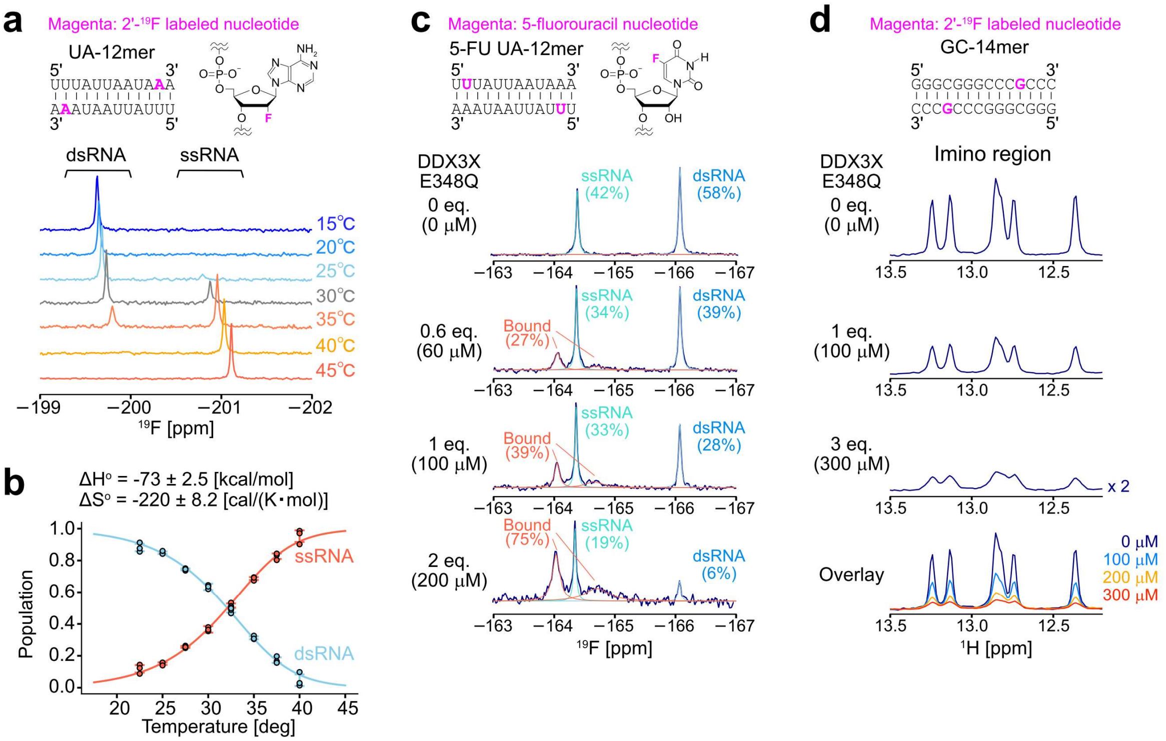

Supplementary Figure 8 ^{19}F and ^{1}H NMR analyses of RNA. (a) ^{19}F 1D spectra of UA-12mer where the 11th adenosine is labeled with ribose 2′-^{19}F measured at various temperatures. The RNA concentration was 100 µM (as a single strand). NMR measurements were performed at 600 MHz. (b) Plots of the fractional population of dsRNA (blue) and ssRNA (orange-red) states of UA-12mer as a function of temperature. The dots represent the experimentally obtained data points, and the lines represent the best-fit curve. Error bars represent the standard deviation of three independent measurements (n=3). The center of the error bar represents the average value. (c) ^{19}F 1D spectra of 5-fluorouracil (FU) UA-12mer where the 2nd uracil is labeled with 5-FU in the presence of varying concentrations of the E348Q variant of DDX3X in the ATP-bound form. The experimentally obtained spectra are shown as navy lines, and deconvoluted lines of dsRNA (blue), ssRNA (turquoise), and bound (orange-red) signals are overlayed. In the 5-FU probe, two bound signals were observed at -164.0 ppm and -164.7 ppm. We interpreted that these two signals reflect differences in the microenvironment of the 5-FU probe depending on the position of DDX3X along the ssRNA chain (*i.e.* whether the binding site of DDX3X is closer to the 5′- or 3′-end in the complex). The RNA concentration was 100 µM (as a single strand). NMR measurements were performed at 30 °C and 600 MHz. (d) Imino ^{1}H 1D spectra of GC-14mer where ribose 2′-^{19}F was introduced to the 11th guanosine in the presence of varying concentrations of the E348Q variant of DDX3X in the ATP-bound form. The overlay of the spectra is shown at the bottom. NMR measurements were performed at 35 °C and 600 MHz using a water-flip-back watergate sequence. Source data are provided as a Source Data file.

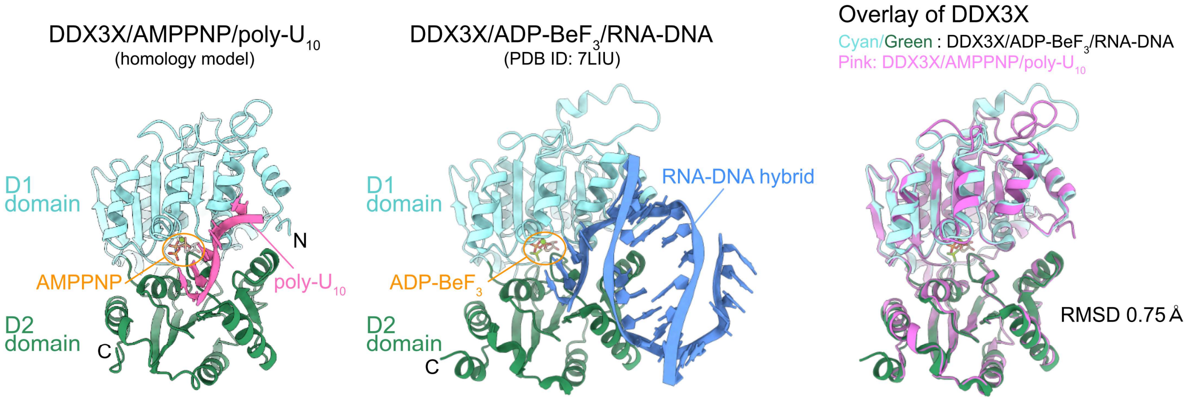

Supplementary Figure 9 Structure of DDX3X in complex with remodeled RNA. Ribbon diagram of the homology model of DDX3X/AMPPNP in complex with poly-U_{10} (left) and DDX3X/ADP-BeF_3 in complex with an RNA-DNA hybrid (5′-r(GGGCGGG)d(CCCGCCC)-3′, where "r" and "d" represent the RNA and DNA nucleotides, respectively) (PDB ID: 7LIU) (center). Only one DDX3X molecule in the unit is shown for clarity. (right) The overlay of the DDX3X structure (cyan/green: DDX3X/ADP-BeF_3/RNA-DNA, pink: DDX3X/AMPPNP/poly-U_{10}). The RMSD value over the pruned set of pairs is shown.
