# Supplementary material for: NMR characterization of RNA binding property of the DEAD-box RNA helicase DDX3X and its implications for helicase activity
Source: Nat Commun. 2024 Apr 25;15:3303. doi: 10.1038/s41467-024-47659-w (PMC11045745; doi:10.1038/s41467-024-47659-w)
Supplement: Supplementary file 3 — Description of Additional Supplementary Information [file 41467_2024_47659_MOESM3_ESM.pdf]

## Description of additional supplementary files

**Supplementary Data 1**: DNA sequence of DDX3X constructs and primers
