# Supplementary material for: NMR characterization of RNA binding property of the DEAD-box RNA helicase DDX3X and its implications for helicase activity
Source: Nat Commun. 2024 Apr 25;15:3303. doi: 10.1038/s41467-024-47659-w (PMC11045745; doi:10.1038/s41467-024-47659-w)
Supplement: Supplementary file 5 — Reporting Summary [file 41467_2024_47659_MOESM5_ESM.pdf]

nature portfolio

Corresponding author(s): Yuki Toyama, Ichio Shimada

Last updated by author(s): Mar 18, 2024

Reporting Summary

Nature Portfolio wishes to improve the reproducibility of the work that we publish. This form provides structure for consistency and transparency in reporting. For further information on Nature Portfolio policies, see our Editorial Policies and the Editorial Policy Checklist.

Statistics

For all statistical analyses, confirm that the following items are present in the figure legend, table legend, main text, or Methods section.

n/a	Confirmed	
☐	☒	The exact sample size (n) for each experimental group/condition, given as a discrete number and unit of measurement
☐	☒	A statement on whether measurements were taken from distinct samples or whether the same sample was measured repeatedly
☒	☐	The statistical test(s) used AND whether they are one- or two-sided<br>*Only common tests should be described solely by name; describe more complex techniques in the Methods section.*
☒	☐	A description of all covariates tested
☒	☐	A description of any assumptions or corrections, such as tests of normality and adjustment for multiple comparisons
☐	☒	A full description of the statistical parameters including central tendency (e.g. means) or other basic estimates (e.g. regression coefficient) AND variation (e.g. standard deviation) or associated estimates of uncertainty (e.g. confidence intervals)
☒	☐	For null hypothesis testing, the test statistic (e.g. F, t, r) with confidence intervals, effect sizes, degrees of freedom and P value noted<br>*Give P values as exact values whenever suitable.*
☒	☐	For Bayesian analysis, information on the choice of priors and Markov chain Monte Carlo settings
☒	☐	For hierarchical and complex designs, identification of the appropriate level for tests and full reporting of outcomes
☒	☐	Estimates of effect sizes (e.g. Cohen's d, Pearson's r), indicating how they were calculated

Our web collection on statistics for biologists contains articles on many of the points above.

Software and code

Policy information about availability of computer code

Data collection	TopSpin 3.5.7 or 4.2.0 (Bruker) for NMR data collection.
Data analysis	NMRPipe 11.1 for NMR data processing. NMRFAM-SPARKY 1.470 for NMR data analyses. Peakipy software package (https://github.com/j-brady/peakipy) (version 0.1.30) for peak integrations. TITAN 1.6 for 2D NMR lineshape analyses. CS Analyzer 4 2.4.5 for gel image analyses. PDB2PQR 3.6.1 and APBS 3.4.1 for the surface charge calculation. WynHydroPRO 1.00 for rotational correlation time calculations. The structures were rendered by using UCSF Chimera X 1.6.1. The duplex stability was estimated by the nearest-neighbor approach using the parameters in 1.0 M NaCl (https://www.konan-fiber.jp/hp/sugimoto/contents/NN/NearestNeighborCalculator.htm). The structure of DDX3X bound to poly-U10 was modeled using the SWISS-MODEL web server (https://swissmodel.expasy.org/) on Sept. 20, 2022. The Python scripts used in this study are available on https://github.com/YukiToyama/RNA_binding_property_of_DDX3X (http://dx.doi.org/10.5281/zenodo.10826902).

For manuscripts utilizing custom algorithms or software that are central to the research but not yet described in published literature, software must be made available to editors and reviewers. We strongly encourage code deposition in a community repository (e.g. GitHub). See the Nature Portfolio guidelines for submitting code & software for further information.

Data

Policy information about availability of data

All manuscripts must include a data availability statement. This statement should provide the following information, where applicable:

- Accession codes, unique identifiers, or web links for publicly available datasets
- A description of any restrictions on data availability
- For clinical datasets or third party data, please ensure that the statement adheres to our policy

NMR assignments for the D1 and D2 domains of DDX3X have been deposited to the BMRB database under accession numbers 52143 [https://doi.org/10.13018/BMR52143] and 52142 [https://doi.org/10.13018/BMR52142], respectively. Structure data used in this study are available in the Protein Data Bank under accession codes 1RNA [https://doi.org/10.2210/pdb1RNA/pdb], 2DB3 [https://doi.org/10.2210/pdb2DB3/pdb], 2KOC [https://doi.org/10.2210/pdb2KOC/pdb], 5E7I [https://doi.org/10.2210/pdb5E7I/pdb], 5E7J [https://doi.org/10.2210/pdb5E7J/pdb], 5E7M [https://doi.org/10.2210/pdb5E7M/pdb], and 7LIU [https://doi.org/10.2210/pdb7LIU/pdb]. The protein sequence used in this study is available from the UniProt database under accession code O00571 (DDX3X) [https://www.uniprot.org/uniprotkb/O00571/entry]. Source data are provided with this paper.

Research involving human participants, their data, or biological material

Policy information about studies with human participants or human data. See also policy information about sex, gender (identity/presentation), and sexual orientation and race, ethnicity and racism.

Reporting on sex and gender	N/A
Reporting on race, ethnicity, or other socially relevant groupings	N/A
Population characteristics	N/A
Recruitment	N/A
Ethics oversight	N/A

Note that full information on the approval of the study protocol must also be provided in the manuscript.

Field-specific reporting

Please select the one below that is the best fit for your research. If you are not sure, read the appropriate sections before making your selection.

☒ Life sciences ☐ Behavioural & social sciences ☐ Ecological, evolutionary & environmental sciences

For a reference copy of the document with all sections, see nature.com/documents/nr-reporting-summary-flat.pdf

Life sciences study design

All studies must disclose on these points even when the disclosure is negative.

Sample size	No statistical methods were used to predetermine the sample size. For all the data sets provided in this work, we chose the replicate size based on our own preliminary experiments, which focused on the magnitude and consistency of measurable differences.
Data exclusions	The data were not excluded from the analyses
Replication	All attempts at replication were successful. The numbers of experiments are described in the Methods section or figure legend, where applicable.
Randomization	Randomization method was not utilized. This was not a clinical trial (i.e., no animals or humans were involved) and different bacterial strains were not being compared to each other.
Blinding	Blinding is not relevant for this study because no group allocation was performed.

Reporting for specific materials, systems and methods

We require information from authors about some types of materials, experimental systems and methods used in many studies. Here, indicate whether each material, system or method listed is relevant to your study. If you are not sure if a list item applies to your research, read the appropriate section before selecting a response.

Materials & experimental systems

n/a	Involved in the study
☒	☐ Antibodies
☒	☐ Eukaryotic cell lines
☒	☐ Palaeontology and archaeology
☒	☐ Animals and other organisms
☒	☐ Clinical data
☒	☐ Dual use research of concern
☒	☐ Plants

Methods

n/a	Involved in the study
☒	☐ ChIP-seq
☒	☐ Flow cytometry
☒	☐ MRI-based neuroimaging

Plants

Seed stocks	N/A
Novel plant genotypes	N/A
Authentication	N/A
